# One health rapid qualitative assessment: Exploring local governance gaps in Tanzania

**DOI:** 10.1016/j.onehlt.2026.101353

**Published:** 2026-02-10

**Authors:** Olivier Rubin, Suzana S. Nyanda, Madelaine Norström

**Affiliations:** a Department of Social Sciences and Business, Roskilde University, Denmark; b Department of Policy, Planning and Management, Sokoine University of Agriculture, Tanzania; c Department of Animal Health, Welfare and Food Safety, Norwegian Veterinary Institute, N-1433 Ås, Norway

**Keywords:** Rapid qualitative assessment, Strategy–practice gap, Subnational OH governance, Implementation barriers, Tanzania

## Abstract

The One Health Approach (OHA) is globally recognized as essential for addressing complex health threats such as antimicrobial resistance (AMR). However, translating national commitments into coordinated subnational practice remains a major governance challenge. This paper presents the OH Rapid Qualitative Assessment (OH-RQA) as a rapid, resource-efficient diagnostic tool for analyzing subnational implementation. Using five analytical dimensions (thinking, planning, working, sharing, and learning) the OH-RQA examines how practitioners and implementers at the local level understand and apply the OHA.

We applied the OH-RQA in two Tanzanian regions in the context of AMR, collecting data through focus group discussions and semi-structured interviews with district officials in human, animal, and environmental sectors. Findings reveal persistent gaps between national OHA strategies and local governance practices, including limited cross-sectoral coordination, inconsistent awareness of national AMR priorities, and structural barriers to integrated action. Officers identified absent policy mandates, insufficient guidance from higher administrative levels, lack of training and incentives, and the exclusion of OHA from most job descriptions as key obstacles.

By providing a scalable, structured, qualitative diagnostic framework focused on local practice, the OH-RQA supports targeted recommendations for strengthening subnational AMR governance. Beyond Tanzania, it offers a transferable approach for rapidly assessing OH implementation across diverse crisis governance contexts, including pandemic preparedness, climate adaptation, and cross-border health threats.

## 1 Introduction

The One Health Approach (OHA) has been established as a successful global public health initiative that is internationally recognized as best practice to address key public health issues such as zoonotic diseases and antimicrobial resistance (AMR) [1,2]. The OHA has spread to most corners of the world including the Global South. Several countries in Sub-Saharan Africa have published dedicated One Health (OH) national action plans, and many more have included OHAs in their National Action Plans on AMR [[3], [4], [5]]. In this paper, “One Health” (OH) refers to the overarching concept of integrated, multisectoral action on human, animal, and environmental health while “One Health Approach” (OHA) refers to the operational application of OH principles in planning, policy, and implementation. In short, the OHA seeks to optimize the health of humans, animals and ecosystems by integrating these fields rather than keeping them separate [6]. Such a holistic public health approach calls for communication, coordination, and collaboration between the relevant sectors and disciplines on many levels [7]. Nearly 90% of countries from the African region report that all three sectors, (e.g., human, animal and the environment) are actively involved in multisector coordination of OH initiatives on AMR [8]. Still, several studies report a discrepancy between successful national strategic initiatives and then actual implementation on the ground [[9], [10], [11], [12]]. The Global One Health Index, which scores countries from 0 to 100 based on approximately 50 indicators, also reveals stark disparities: in 2022 the scores ranged from 29 in Guinea-Bissau to 71 in the United States [13,14]. An OHA assessment focusing on Sub-Saharan Africa by Fasina et al. [15] found considerable variation in OH integration even within Sub-Saharan African countries with institutional scores ranging from 0.2 to 0.7 (on a 0–1 scale). These findings underscore a key challenge: while most governments formally endorse the OHA and report strong intersectoral collaboration at the national level, significant discrepancies persist in translating these commitments into effective, coordinated action at the subnational and institutional levels. This implementation gap is increasingly recognized as a global challenge, particularly in low- and middle-income countries where subnational actors are pivotal for operationalizing national OHA commitments.

Given the persistent gap between strong national-level commitment to the OHA and more limited implementation at the subnational level, this paper pursues a dual objective.

Methodologically, it introduces and outlines an OH Rapid Qualitative Assessment tool (OH-RQA) that can provide rapid and context-specific insights into subnational OHA operationalization and implementation. Rather than relying on conventional public health metrics and outcome indicators, the OH-RQA focuses on the practical application of core OHA principles, multisectoral coordination and collaboration, at the subnational level. By conceptualizing OH as an *approach* rather than an *outcome*, the OH-RQA offers a replicable, resource-efficient means of assessing the penetration of OHA at the local level and facilitates context-specific analysis of best practices and barriers to OHA implementation.

Empirically, the paper presents findings from an OH-RQA in Tanzania. Tanzania is an interesting case due to its high level of government commitment to the OHA in key public health challenges such as zoonotic diseases and AMR. It stands out as the only country in the region to have produced two national action plans on AMR that explicitly emphasize the OHA *as well as* two dedicated National Strategies on OH [[16], [17], [18], [19]]. Moreover, in the assessment by Fasina et al. [15], Tanzania's National OH Platform received the highest score of comparable platforms in Uganda, Kenya, Malawi and Mali, and a perfect score (100%) in the domain of working practices such as collaboration, flexibility, adaptability, and transdisciplinarity [18]. Thus, Tanzania can be characterized as having a strong national commitment to advancing the principles of the OHA. However, findings from the OH-RQA reveal that, despite Tanzania's strong national-level commitment, the OHA is only partially realized at the subnational level. Other recent Tanzanian studies on OH subnational implementation have reached similar conclusions [[20], [21], [22], [23]]. This only underscores the critical need to shift greater analytical and policy attention to local-level dynamics and to better understand the challenges many countries in the Global South face in translating high-level OHA commitments into local governance practice: why has Tanzania's strong national OHA commitments not translated into local action?

## 2 Methods

### 2.1 OH-RQA's analytical dimensions

The OH-RQA is a qualitative tool to assess adherence to the governance principles of OHA at the subnational level. The approach builds on a key component of the Network for Evaluation of One Health (NEOH), namely the elements associated with measuring “OH-ness” in the implementation and infrastructure of the OHA: (i) OH thinking; (ii) OH planning; (iii) OH working; (iv) OH sharing and (v) OH learning [24]. The OH-RQA maps these qualitative insights directly onto the five “OH-ness” elements (Table 1), and the semi-structured interview guide is provided in the supplementary material for replication. Table 1 outlines the five analytical dimensions of OH-RQA with their associated domains, focus and exemplary questions.

**Table 1:** . OH-RQA analytical dimensions and examples of guiding questions.

NEOH dimension	Domain	Focus	Question example
*Thinking*	Knowledge	Awareness of OHA	“Have you ever heard of the OHA”?
*Planning*	Practices	Participation in planning processes of OHA	“Have you been involved in any planning activities for OH initiatives?”
*Working*	Practices	Collaboration across sectors/agencies	“Do you collaborate with other sectors?”
*Sharing*	Practices & knowledge	Information/reporting behavior across sectors	“How is public health information shared between sectors/agencies?”
*Learning*	Practices & knowledge	Training in OHA	“Have you received any training related to OHA?”

Importantly, whereas the NEOH's evaluation framework provides a multidimensional quantitative assessment of distinct OHA initiatives at the system level [24,25], the OH-RQA qualitatively assesses OHA knowledge and practices at the local governance level. Unlike NEOH, the OH-RQA is designed for rapid deployment, requires minimal resources, and focuses specifically on district-level implementation rather than national commitment. This makes it better suited for diagnosing practical AMR governance challenges in settings where subnational actors are the key operational link between national policy commitments and on-the-ground implementation. Furthermore, OH-RQA's qualitative diagnostic approach prioritizes capturing lived experiences and implementation bottlenecks over producing a composite numerical score. This enables more targeted recommendations in resource-constrained environments where AMR implementation relies heavily on subnational capacity.

### 2.2 The case-context

This OH-RQA was conducted as part of a larger research project on AMR with participating researchers from both Tanzania and Europe, *Interventions for improved knowledge and awareness of antimicrobial resistance using a One Health approach in Tanzania*, which addresses AMR in poultry food production across the two regions of Kilimanjaro and Mwanza (partly supported by the Research Council of Norway's Global Health Initiative, project number 336223). One of the key deliverables of the project was to assess how the local government in these two regions applied OHA when dealing with AMR in livestock production. Hence, the OH-RQA was applied in both regions through two one-day workshops in Mwanza (November 16, 2023 and May 2, 2025) and Moshi (November 13, 2023 and April 29, 2025), respectively. Collecting data in two districts at two different periods increased analytical robustness and allowed for a comparative perspective but the OH-RQA is also well-suited as a stand-alone exercise at one point in time.

### 2.3 OH-RQA data collection

Data collection primarily relied on focus group discussions and selected semi-structured interviews during the one-day workshops. Participants were selected by the authors in coordination with local government authorities to ensure those with responsibilities in AMR or zoonotic disease control were invited. This purposive sampling targeted the key sectors of the OHA at the district level: human, animal and the environment (see Table 2).

**Table 2:** Participants overview in the workshops, Mwanza and Kilimanjaro, 2023 and 2025.⁎

Focus Group Discussions			
Region	Year	Participant Category	Details
Mwanza	2023	Environmental Officers	3 from Magu, Nyamagana, and Ilemela Districts
Mwanza	2023	Veterinary Officers	3 from Magu, Nyamagana, and Ilemela Districts
Mwanza	2023	Medical & Education Officers*	3 medical officers and 2 education officers from Magu and Nyamagana Districts
Mwanza	2025	Environmental Officers	3 from Magu, Nyamagana, and Ilemela Districts
Mwanza	2025	Veterinary Officers	3 from Magu, Nyamagana, and Ilemela Districts
Mwanza	2025	Medical Health Officers	3 from Magu, Nyamagana, and Ilemela Districts
Kilimanjaro	2023	Environmental Officers	3 from Moshi DC, Moshi MC, and Hai DC
Kilimanjaro	2023	Veterinary Officers	3 from Moshi DC, Moshi MC, and Hai DC
Kilimanjaro	2023	Medical & Education Officers	3 medical officers and 2 education officers from Moshi DC and Moshi MC
Kilimanjaro	2025	Environmental Officers	3 from Moshi DC, Moshi MC, and Hai DC
Kilimanjaro	2025	Veterinary Officers	3 from Moshi DC, Moshi MC, and Hai DC
Kilimanjaro	2025	Medical Health Officers	3 from Moshi DC, Moshi MC, and Hai DC
Kilimanjaro	2025	Ministry Officials	National Coordinator (PMO), National Epidemiologist (Livestock), AMR Coordinators (Human and Livestock sectors)


Key Informant Interviews			
Region	Year	Participant Category	Details
Mwanza	2025	Key Informant Interviews	2 veterinary officers, 2 environmental officers, 1 medical officer (from Nyamagana, Ilemela, and Magu DCs)
Kilimanjaro	2025	Key Informant Interviews	National Coordinator (PMO), National Epidemiologist (Livestock sector), National AMR Coordinator

⁎ Education officers were included within the health sector category due to their role in community health awareness, health education, and AMR-related outreach.

Each workshop brought together around 15–20 district officers. These were then divided into three smaller focus groups of 3–6 individuals from the same sector (e.g., human, animal, or environmental health). Three focus group discussions were run in parallel and were facilitated by one moderator and a dedicated note-taker. The discussions lasted approximately 45–60 min, and most were conducted in Swahili to ensure participant comfort and facilitate discussions. Data collection in the second round was done in a larger group composed of all participants from the three sectors (human, animal, or environmental health). The aim was to collect additional data based on the preliminary findings from the first round of data collection. During this round, follow-up semi-structured interviews (15–30 min) were conducted with selected participants based on focus group discussion observations to expand on emerging themes. Interviews followed the same core guide as the focus group discussions. All participants provided informed consent orally; discussions were conducted under assurances of confidentiality and anonymity. The informed consent template has been uploaded in the supplementary material. A gender imbalance was noted with approximately 75% of participants identifying as men, but this appears to be reflective of the broader gender-composition of district-level officials [26,27].

### 2.4 OH-RQA data categorization

Data categorization was based primarily on detailed notes from the focus group discussions and interviews, which were translated from Swahili into English. All notes were anonymized by removing names and other identifiable information. Two authors independently and iteratively categorized the notes according to the five OH-RQA dimensions. Original audio recordings were consulted and translated when clarification or additional nuance was required. Minor differences in categorization were discussed and reconciled until consensus was reached, ensuring consistency. Key themes and illustrative quotes were then extracted for each dimension. By applying a deductive framework with only five predefined categories, the data could be structured and categorized without the use of specialized qualitative software. With over 30 district officials participating in each region from different sectors, thematic saturation appears to have been reached across all five dimensions, as there was a high degree of thematic overlap between the focus group discussions and no significant variation or elaboration beyond the predefined OH-RQA categories [28].

## 3 Results

### 3.1 OHA thinking

The focus group discussions revealed that OHA thinking was not a prominent feature in the day-to-day work of district officers. Across both regions, most participants (particularly the environmental officers) stated they had limited awareness of the concept and the principles underlying it. An environmental officer stated: *“I had never heard of the One Health approach before this workshop”* (2023, Environmental Officer, Mwanza) while a medical health officer admitted to having seen the strategic plan *“but never actually read it”* (2023, Medical Officer, Kilimanjaro). Others reported having heard of the term, but that they were unsure of its overarching meaning and practical relevance. Even among those familiar with the concept, OHA was rarely integrated as a guiding framework in their routine responsibilities. Instead, human, animal, and environmental health concerns were typically addressed within separate sectoral mandates. As one veterinary officer explained: “Every sector has its own responsibility. For example, a medical officer has the mandate to close the butcher if the butcher has no health clearance even though the meat sold has no problem” (2023, Veterinary Officer, Kilimanjaro). In such cases, the medical officer focuses on enforcing health clearance requirements while the veterinary officer's priority is the safety and quality of the meat. Each of them acts within their own legal mandate which leads to parallel rather than coordinated OHA responses.

### 3.2 OHA planning

Given the generally low awareness of the OHA, formal planning based on OHA principles was also limited. Most intersectoral collaborations appeared to be reactive rather than strategic, occurring mainly in response to emergencies (such as rabies or anthrax outbreaks) rather than as part of routine joint planning. District officers described how coordination between human, animal, and environmental sectors was typically initiated only when a concrete problem arose. A veterinary officer noted: *“We don't really plan together; we only meet when there's an issue”* (2023, Veterinary Officer, Mwanza). Environmental officers, despite their relevance to zoonotic disease prevention and AMR management, were most frequently not involved in any planning, which contributed to sectoral silos in addressing AMR.

Participants also pointed to structural barriers, such as a lack of dedicated budgets, weak health infrastructure, and limited laboratory capacity, that together impede both preparedness and collaboration and undermine strategic planning. As one medical health officer explained: *“There are no laboratories that conduct culture and sensitivity tests. If this was available, it would solve the problem about 80%, but it is still lacking at the local government level”* (2023, Medical Officer, Kilimanjaro). Disaster preparedness plans were among the joint activities mentioned; however, inadequate budgets remained a challenge: *“we usually do not allocate funds for disasters until they start happening; that's when we start looking for money”* (2023, Medical Officer, Mwanza).

### 3.3 OHA working

Concrete practices reflecting OHA were limited at the subnational level. While some collaboration occurred during specific events such as rabies outbreaks, these were exceptions rather than the norm. One veterinary officer explained: *“Do we work with human and environmental sectors on OH? Rarely. But we work together with the human health sector in situations like rabies”* (2023, Veterinary Officer, Mwanza). Overall, cross-sectoral relationships were fragmented and reliant on informal channels or personal initiative rather than institutional routines. Collaboration was reactive and event-driven, with environmental officers often marginal to OHA activities. As one explained: *“In my job description, there are no responsibilities on AMR or OHA. I was asked to represent the district environmental officer one day before this workshop without being informed of the objective”* (2025, Environmental Officer, Mwanza). This limited collaboration was compounded by unclear mandates and resource constraints. A medical officer linked siloed working directly to governance and funding arrangements: *“Why are we working in silos? Because of the policy we have as well as the financial constraints”* (2023, Medical Officer, Mwanza). Similarly, a ministry official described budgetary barriers: *“It is very difficult to get budget allocation on AMR activities since it is an emerging health challenge and given low priority compared with other disease outbreaks”* (2025, Ministry Official, Kilimanjaro). Thus, participants across sectors expressed support for OHA in principle but found few incentives and limited structural support for embedding it in daily workflows or planning.

### 3.4 OHA sharing

Information and knowledge sharing across sectors was limited and largely ad hoc. While medical and veterinary health officers occasionally exchanged information, there was minimal structured engagement to institutionalize these exchanges. Environmental officers were especially marginal to such processes. District officers described an institutional culture in which different departments operated in parallel with little interaction. As one environmental officer remarked: *“We work in the same building, but I didn't even know what the others were doing. This is the first time I meet some of the other officers”* (2023, Environmental Officer, Kilimanjaro). Even among veterinary and medical officers, knowledge sharing on OHA was uneven and informal. A veterinary officer explained: *“We do communicate as colleagues or friends, but not at an institutional level”* (2023, Veterinary Officer, Mwanza). A ministry official expanded on these observations, noting that institutional structures for AMR control through OHA do exist, but they are not widely communicated or recognized at the district level (2025, Ministry Official, Kilimanjaro). This lack of structured and widely understood communication mechanisms undermined OHA implementation in practice. Although proximity of different departments might suggest potential for collaboration, the absence of fora for knowledge sharing and institutionalized communication channels reinforced siloed working patterns.

### 3.5 OHA learning

Opportunities for structured learning about the OHA appeared scarce at the subnational level. A veterinary officer explained: *“the only knowledge I have is from university, and I haven't had any training since then”* (2023, Veterinary Officer, Kilimanjaro). While some district officers had participated in past OHA trainings, often linked to other public health priorities such as food safety, these were the exception rather than the norm. Even among those familiar with the OH term, few had received dedicated training. One medical officer noted that he had recently attended a OH training workshop in 2024 but that *“it was just a one-off event”* (2025, Medical Officer, Kilimanjaro). Again, the lack of training was most prominent in the environmental sector where officers noted the lack of technical guidance, regulatory frameworks, and policy documents connecting environmental health issues to OHA and AMR. Despite these constraints, most participants were generally open to learning about OHA. However, participants emphasized that this would require the establishment of appropriate structures and incentives such as higher-level guidance and more targeted OHA-focused training. A veterinary officer noted that he had learned about successful OHA efforts in Uganda and Kenya during a master's program but that such efforts had not been shared in a Tanzanian context. Participants frequently pointed to other successful public health campaigns like HIV/AIDS and malaria as evidence that coordinated, well-supported efforts have previously worked, and that it might work for OHA and AMR as well.

In summary, the OH-RQA revealed that the OHA is only sporadically recognized and applied at the local level. Awareness is generally limited, cross-sector planning is mostly reactive and siloed, information sharing is partial, and systematic training is lacking. These findings align with patterns generally observed in other decentralized and resource-constrained governance contexts, suggesting that the barriers identified are not unique to this setting but may reflect broader challenges in operationalizing OHA at subnational levels globally [14].

## 4 Discussion

The core rationale behind the OH-RQA tool is that effective implementation of OHA ultimately depends on local-level awareness and implementation. Unlike national composite indicators or global indices, qualitative tools like the OH-RQA allow for assessments of OHA practices on the ground. The OH-RQA not only generates context-specific insights but also raises awareness among participants. Through the focus group discussions in 2023, many district officers were introduced, some for the first time, to the core principles of the OHA. The OH-RQA also carries some limitations. First, participants were selected through authority nominations, which may have introduced a degree of selection bias toward more engaged or knowledgeable officers. Second, as data were translated from Swahili into English, some loss of nuance may have occurred. Beyond these methodological considerations, the tool is relatively resource-efficient and easy to apply, although it does demand effective facilitation of the discussions and overcoming language barriers. As a stand-alone tool, the rapid qualitative approach means findings are ultimately context-specific with limited generalizability across countries. Further, the findings are also primarily rooted in reported behavioral insights and do not include any systematic examination of budget and policy documents. The identified institutional and budgetary barriers to OHA, therefore, were inferred from the district officers' perception. The tool might also suffer from social desirability or courtesy bias in the focus group discussions where the district officers might have downplayed or exaggerated certain practices in front of their colleagues and researchers [29]. However, the frequent and candid admissions of limited awareness and understanding of the OHA suggest that such bias was likely limited in this context. Still, triangulation with additional outcome data appears beneficial. The OH-RQA provides a nuanced snapshot of OHA implementation that can both function as a steppingstone for further analysis or as a contained study of concrete OHA knowledge and practice at the local level. As such, its structure and methodological simplicity make it applicable in a wide range of governance settings where national OHA commitments need to be assessed at the local level. Research could, for example, triangulate OH-RQA findings with quantitative data such as measuring actual interdepartmental communication or analyzing budget allocations for OH activities at district level.

Applying this bottom-up OH-RQA in two regional contexts revealed a range of governance challenges that continue to hinder subnational OHA implementation. Most notably, the OH-RQA identified a clear disconnect between national strategies of the OHA and local-level execution. A particularly striking finding was the limited awareness of OHA among local officials, even among those working in sectors most relevant to zoonotic disease and AMR. Several officers were unfamiliar with core OHA strategic documents, and this lack of awareness extended beyond conceptual understanding to practical application. The complexity of *both* OHA and AMR requires that local officials not only understand each concept individually (which is difficult enough) but also how they intersect. This dual demand for internalizing two complex global concepts (OHA and AMR) places significant cognitive and operational burdens on local officials. Consistent with prior studies [22,23,30], the environmental health sector emerged as the most difficult to integrate into OHA practices. Environmental officers rarely saw AMR or OHA as part of their core mandate, nor was it embedded in their job descriptions or reporting structures.

These findings reinforce a central tenet from public administration research: knowledge and awareness alone are rarely sufficient for sustained institutional change. The traditional *principal–agent theory* offers a useful lens for understanding the disconnect between national policymakers (principals) and district officials (agents) in implementing the OHA [31,32]. Informational asymmetries (such as the widespread unawareness of OHA among district officers) as well as incentive misalignments (where mandates, budgets, and performance indicators are tied to sector-specific tasks rather than OHA) lead district officials to prioritize routine responsibilities over broader intersectoral collaboration. This also ties to another public administration theory of *street-level bureaucracy* emphasizing that public policy is not just determined by national strategies but shaped by the actions and judgments of the local public servants (“street-level bureaucrats”) who implement it [33,34]. In many contexts, especially where AMR and OHA are externally promoted and conceptually demanding, local officials may lack organizational support, incentives, policy clarity, and accountability mechanisms to internalize these concepts in practice. Rather than a lack of willingness, the limited uptake may reflect a form of “agenda conformity” [35] or “decoupling” [36] where local actors are tasked with implementing global health agendas without the structural or motivational scaffolding necessary to succeed. These understandings help shift the focus from individual-level awareness to the broader governance conditions under which OHA is implemented.

National OH Strategies in several countries now mandate the establishment of multisectoral technical committees at the subnational level. Embedding OHA in job descriptions, ensuring dedicated budgets for OHA training, mandating meeting frequencies across sectors, and linking performance reporting to collaborative activities would not only align with the OH national strategies but also address some of the key implementation gaps identified in this study. Concretely, regular intersectoral meetings could be made mandatory or officers could be required to document joint responses to public health issues such as combatting AMR. While such requirements would add to workloads, they are relatively cost-effective and could foster long-term institutional synergies and local ownership of the OHA. Such measures would help in moving OHA beyond a strategic national vision to become a practiced reality at the district level.

## 5 Conclusion

In conclusion, the OH-RQA tool proved effective in rapidly revealing how national strategies of relevance for OHA are taking root in local governance. The rapid qualitative assessment in Tanzania revealed that strong national OH commitments have yet to fully permeate local governance. District officials often lacked awareness of the OHA, worked in silos, and engaged in cross-sector collaboration only during emergencies. This suggests that the OHA is far from institutionalized in day-to-day governance in the districts. The challenges identified appear to stem less from individual reluctance and more from underlying structural and institutional constraints. Integrating the OHA into local practice does not necessarily require large financial investments, but it does require sustained, deliberate efforts such as formally integrating OHA responsibilities into job descriptions and creating meaningful incentives for cross-sector collaboration. Strengthening local-level operationalization should be a central priority in advancing the OHA globally.

## CRediT authorship contribution statement

**Olivier Rubin:** Writing – original draft, Validation, Methodology, Investigation, Formal analysis, Conceptualization. **Suzana S. Nyanda:** Writing – original draft, Validation, Methodology, Formal analysis, Data curation, Conceptualization. **Madelaine Norström:** Writing – review & editing, Project administration, Funding acquisition, Data curation, Conceptualization.

## Ethical approval

Ethical clearance for the project and associated interviews was obtained from the Norwegian Regional Committees for Medical and Health Research Ethics (protocol number 700096) and the National Institute for Medical Research (NIMR), Tanzania.

## Funding

This work was partly supported by the Research Council of Norway's Global Health Initiative (GLOBVAC3), project number 336223.

## Declaration of competing interest

The authors declare no competing interests.

## Data Availability

No data was used for the research described in the article.

## Appendix A Supplementary data

Interview guide and informed consent template. Image 1
