# Supplementary material for: One health rapid qualitative assessment: Exploring local governance gaps in Tanzania
Source: One Health. 2026 Feb 10;22:101353. doi: 10.1016/j.onehlt.2026.101353 (PMC12924893; doi:10.1016/j.onehlt.2026.101353)
Supplement: Supplementary file 1 — Interview guide and informed consent template. [file mmc1.docx]

**Supplementary Material**

1. **Interview Guide – Focus Group Discussions on One Health (OH)**

Note: *This guide was used for semi-structured focus group discussions across the three sectors: human health, animal health, and environmental health. The present version represents a subset of questions drawn from the full interview guide, specifically those that were relevant to OH. The sequence of questions varied between discussions, though most began with awareness-related topics before transitioning to practice-oriented questions. The classification into the five dimensions (Thinking, Training, Planning, Working, and Sharing) was applied retrospectively.*

**Awareness**

- How would you describe the current awareness of AMR and OH in your own sector? ***[Thinking]***
- What does OH mean to you? ***[Thinking]***
- Would you say your colleagues are knowledgeable about AMR/OH? [Thinking]
- Could you share any ongoing initiatives or programs related to AMR/OH in your area? ***[Thinking]***
- Have you received any training related to OH/AMR? ***[Training]***
- Are there any educational campaigns or outreach activities about AMR/OH aimed at schools, farmers, or other groups? ***[Training]***
- Are there successful initiatives or strategies that have effectively raised awareness about AMR/OH in your district? ***[Training]***
- What are your future ideas or goals regarding AMR/OH awareness and mitigation in your district? ***[Training]***
- Are you familiar with the Tanzanian National Action Plan on AMR (2023–2028)? ***[Thinking, Planning, Working, Sharing]***
- What are the key issues in the plan? ***[Thinking, Planning, Working, Sharing]***

**Practice**

- Does the national plans guide your work locally? If yes, how? If no, why not? ***[Thinking, Planning, Working, Sharing]***
- What are the main impediments to national strategy implementation and coordination at the local level? ***[Thinking, Planning, Working, Sharing]***
- How is the OH approach integrated in these strategies? ***[Thinking, Planning, Working, Sharing]***
- Are there any specific guidelines or protocols for veterinary/environmental/human health professionals on AMU? ***[Thinking, Planning, Working, Sharing]***
- Have you been involved in any planning activities for OH initiatives? ***[Planning]***
- How do human, animal, and environmental sectors plan and coordinate on AMR? ***[Planning, Working, Sharing]***
- How is the NAP 2023–2028 being implemented? How is OH integrated? ***[Working, Planning, Sharing]***
- Which sectors do you collaborate with in relation to AMR/OH? Give examples. ***[Working, Planning, Sharing]***
- How do you think the collaboration is going? ***[Working]***
- What are the challenges of collaboration among sectors and stakeholders? ***[Working, Planning, Sharing]***
- What would be needed to improve collaboration? ***[Working, Planning, Sharing]***
- How is public health information shared between sectors? ***[Sharing]***
- How is AMR/OH awareness and education currently integrated into healthcare practices and public health campaigns in Tanzania? ***[Sharing]***
- What challenges do you face in raising awareness about AMR among healthcare professionals, patients, and the public? ***[Sharing]***

1. **Informed Consent Template**

This project aims at reducing further development of AMR in Tanzania and improve the health of humans, animals and the environment. In order to achieve its aim, the project will undertake studies on humans, animals, and environment using a One Health Approach. You have been purposively selected to participate in the consultations to provide us with information. We understand that you are involved in matters related to animal, human or environmental health. As such your information will be invaluable to our research project. While discussing, please allow us to record the responses to capture all the information. Your name will be kept confidential, and the information will be used for research purposes and not otherwise shared. This is a discussion: let us know your views on the key issues we want to understand in relation to the work with animal, human or environmental health. The discussion will last for 45-60 minutes.

First, do you agree to participate in this consultation?

If yes, do you agree to be recorded?

We thank you in advance for your cooperation.
